# Supplementary material for: Discovery and Characterisation of Novel Poly-Histidine-Poly-Glycine Peptides as Matrix Metalloproteinase Inhibitors
Source: Biomolecules. 2025 May 12;15(5):706. doi: 10.3390/biom15050706 (PMC12108757; doi:10.3390/biom15050706)
Supplement: Supplementary file 1 [file biomolecules-15-00706-s001.zip › biomolecules-3594049-supplementary.pdf]

Supplementary Materials

We investigated whether pHpG-H5 and pHpG-H7 exhibit similar inhibitory effects. Time-dependent β-casein degradation mediated by MMP-1 was analysed. In the absence of pHpG peptides, β-casein was progressively degraded and fully hydrolysed within 24 hours (Figure S1A). In contrast, pre-incubation with pHpG-H5, pHpG-H7, or pHpG-H9 significantly inhibited MMP-1 activity in hydrolysing β-casein (Figure S1B-D). Notably, pHpG-H7 demonstrated the most pronounced inhibitory effect, suppressing MMP-1 activity as early as 0.5 hours.

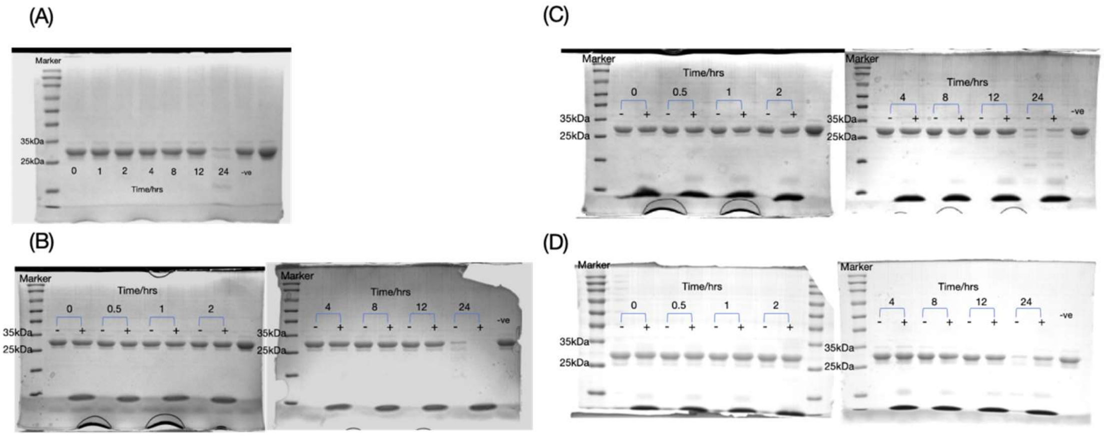

Figure S1. MMP-1 inhibition assay of synthetic pHpG peptides. (**A**) Time-dependent β -casein degradation mediated by MMP-1. Time-dependent β -casein degradation mediated by MMP-1 pre-incubated with pHpG peptides: (**B**) pHpG-H5, (**C**) pHpG-H7, (**D**) pHpG-H9. Molecular mass markers (kDa) in lane M and negative control (vehicle) in lane-ve.
